# Abdominal subcutaneous adipose tissue: a favorable adipose depot for diabetes?

**DOI:** 10.1186/s12933-018-0734-8

**Published:** 2018-06-26

**Authors:** Peizhu Chen, Xuhong Hou, Gang Hu, Li Wei, Lei Jiao, Hongmei Wang, Siyu Chen, Jingzhu Wu, Yuqian Bao, Weiping Jia

**Affiliations:** 1 Department of Endocrinology and Metabolism, Shanghai Jiao Tong University Affiliated Sixth People’s Hospital, 600 Yishan Road, Shanghai, 200233 China; 2 Shanghai Diabetes Institute, Shanghai, China; 3 Shanghai Clinical Center for Diabetes, Shanghai, China; 4 Shanghai Key Laboratory of Diabetes Mellitus, Shanghai, China; 5 Pennington Biomedical Research Center, Baton Rouge, LA USA; 6 Department of Radiology, Shanghai Jiao Tong University Affiliated Sixth People’s Hospital, Shanghai, China

**Keywords:** Subcutaneous adipose tissue, Visceral adipose tissue, Newly diagnosed diabetes, Chinese adults

## Abstract

**Background:**

Previous studies have documented that visceral adipose tissue is positively associated with the risk of diabetes. However, the association of subcutaneous adipose tissue with diabetes risk is still in dispute. We aimed to assess the associations between different adipose distributions and the risk of newly diagnosed diabetes in Chinese adults.

**Methods:**

The Shanghai Nicheng Cohort Study was conducted among Chinese adults aged 45–70 years. The baseline data of 12,137 participants were analyzed. Subcutaneous and visceral fat area (SFA and VFA) were measured by magnetic resonance imaging. Diabetes was newly diagnosed using a 75 g oral glucose tolerance test.

**Results:**

The multivariable-adjusted odds ratios (OR) and 95% confidence intervals (CI) of newly diagnosed diabetes per 1—standard deviation increase in SFA and VFA were 1.29 (1.19–1.39) and 1.61 (1.49–1.74) in men, and 1.10 (1.03–1.18) and 1.56 (1.45–1.67) in women, respectively. However, the association between SFA and newly diagnosed diabetes disappeared in men and was reversed in women (OR 0.86 [95% CI, 0.78–0.94]) after additional adjustment for body mass index (BMI) and VFA. The positive association between VFA and newly diagnosed diabetes remained significant in both sexes after further adjustment for BMI and SFA. Areas under the receiver operating characteristic curve of newly diagnosed diabetes predicted by VFA (0.679 [95% CI, 0.659–0.699] for men and 0.707 [95% CI, 0.690–0.723] for women) were significantly larger than by the other adiposity indicators.

**Conclusions:**

SFA was beneficial for lower risk of newly diagnosed diabetes in women but was not associated with newly diagnosed diabetes in men after taking general obesity and visceral obesity into account. VFA, however, was associated with likelihood of newly diagnosed diabetes in both Chinese men and women.

**Electronic supplementary material:**

The online version of this article (10.1186/s12933-018-0734-8) contains supplementary material, which is available to authorized users.

## Background

Diabetes, an established risk factor of cardiovascular disease (CVD), is one of the fastest growing public health problems in the world [1]. Obesity, especially abdominal obesity, is a well-known underlying risk factor for the development of diabetes [2]. Recent studies suggested that excess adiposity in specific body depots might be associated with different risks of diabetes. For example, abdominal adiposity, in particular, visceral adipose tissue, is positively associated with the risk of diabetes among whites, African Americans, and Japanese Americans [3–5], but the association of subcutaneous adipose tissue from the abdominal or thigh region with diabetes risk is still in dispute [6, 7]. Given the complex nature of measuring depot-specific adipose tissue in living subjects, very few large studies have measured subcutaneous adipose tissue and visceral adipose tissue using magnetic resonance imaging (MRI) or computed tomography (CT) to assess their associations with diabetes risk.

China has the world’s largest diabetes epidemic. According to the latest data, 10.9% of Chinese adults have diabetes [8]. The ranges of body mass index (BMI) of Asian population are different to those of the European or African population. According to an expert consultation from the World Health Organization (WHO), generally, the mean or median BMI is lower in Asian populations than in non-Asian populations [9]. It has been hypothesized that Asians have higher adiposity per unit BMI compared with other ethnic groups, which puts them at an increased risk of type 2 diabetes mellitus at a lower level of BMI [9]. However, very few Chinese studies have assessed the association of different depot-specific adipose tissues with the risk of diabetes. Thus, our aim were to explore the associations between different adipose distributions (subcutaneous adipose tissue and visceral adipose tissue) measured by MRI and the risk of newly diagnosed diabetes screened by an oral glucose tolerance test (OGTT) in a large-sample study of Chinese adults.

## Methods

### Study participants

The Shanghai Nicheng Cohort Study was designed to assess the prevalence, incidence, and related factors of cardiometabolic diseases among adults in Nicheng County, a suburb of Shanghai, China. The baseline survey was conducted between April 2013 and August 2014. The target population covered 23,375 residents aged 45–70 years who had lived in Nicheng County for at least 5 years. A total of 21,408 residents were enrolled, and 17,212 participants finished the baseline survey (mean age 56.9, proportion of Men 45.3%). We excluded the participants without data on BMI, waist circumference or body fat percentage (n = 1091), fasting or 2-h glucose (n = 75), subcutaneous fat area (SFA) and visceral fat area (VFA) (n = 2861), and the participants with a history of diabetes (n = 1048). We excluded the participants with previously diagnosed diabetes because body weight changes have been reported for diabetes treatment in patients with known diabetes. The final sample in the present cross-sectional analysis comprised 12,137 participants (mean age 56.7, proportion of Men 44.5%), the participation flowchart was shown in Additional file 1. The study was approved by the Independent Ethics Community of Shanghai Sixth People’s Hospital and written informed consent was obtained from each participant.

### Baseline measurements

Information on demographics, family history of diseases, medical history, leisure-time physical activity, smoking habits, and alcohol consumption was obtained through a standard questionnaire by trained investigators at local community clinics. Based on responses, the participants were classified as never smokers, ex-smokers, and current smokers. The participants was categorized as never, past, and current drinkers according to their alcohol consumption. The participants reported their leisure-time physical activity as 0, 1–29 min/day, and ≥ 30 min/day. Education was grouped as primary school or less, middle school, and high school or more. Family history of diabetes was defined as having a first-degree relative with diabetes.

Body weight and height were measured using the standardized protocol by specially trained investigators. Height and weight were measured without shoes and with light clothing. The measurements of height were rounded to the nearest centimeter, and weight to the nearest 100 g. Blood pressure was measured twice from the right arm after 5 min of sitting using a mercury sphygmomanometer at 3-min intervals, and the mean value was calculated. Waist circumference was measured at the midpoint between the lowest rib and the iliac crest on the mid-axillary line. BMI was calculated as weight in kilograms divided by the square of height in meters. Body fat was estimated with a Tanita body composition analyzer (TBF-418, Tanita Corp., Tokyo, Japan). Blood samples were collected from participants after an overnight fast of at least 10 h. Participants without a self-reported history of diabetes were administered an OGTT of 75 g glucose. Blood samples were drawn at 0, 30, and 120 min following the OGTT test. Plasma glucose was assessed by a glucose oxidase method. Glycated hemoglobin A1c (HbA1c) values were measured by high-performance liquid chromatography (VARIANT II, Bio-Rad Laboratories, Inc., Hercules, USA).

### Measurement of abdominal adipose tissue

The day after the first visit for blood sampling and other measurements mentioned above, participants underwent abdominal MRI investigations via a 3.0 T General Electric scanner (GE Healthcare, Milwaukee, WI, USA) equipped with an abdominal coil in the Shanghai Jiao Tong University Affiliated Sixth People’s Hospital. Each participant was positioned supine in the magnet and was scanned in cross-sectional planes. T1 axial images were obtained and centered at the navel with a slice thickness of 10.0 mm for 8 slices. Based on the Framingham Heart Study, Irlbeck et al. found that SFA and VFA at the umbilical level strongly correlated to visceral and subcutaneous fat volumes after adjustment for age in both women and men (r = 0.94 for SFA and subcutaneous fat volume and r = 0.98 for VFA and visceral fat volume in men; r = 0.99 for SFA and subcutaneous fat volume and r = 0.92 for VFA and visceral fat volume in women) [10]. In the present study, SFA and VFA were obtained from the umbilical slice, based on an area of 2-D pixels meeting the adipose shading threshold from the DICOM images of each participant. Segmentation of the images into SFA and VFA was conducted by two trained investigators using sliceOmatic image analysis software (version 5; Tomovision Inc., Montreal, QC, Canada). If results differed by more than 10%, a third investigator who did not know the results reanalyzed the images.

### Definition

According to the 1999 WHO criteria [11], category of glucose regulation was defined as follows using an OGTT: isolated impaired fasting glucose (6.1 mmol/L ≤ fasting plasma glucose [FPG] < 7.0 mmol/L and 2-h plasma glucose [2hPG] < 7.8 mmol/L), isolated impaired glucose tolerance (FPG < 6.1 mmol/L and 7.8 mmol/L ≤ 2hPG < 11.1 mmol/L), combined impaired fasting glucose and impaired glucose tolerance (6.1 mmol/L ≤ FPG < 7.0 mmol/L and 7.8 mmol/L ≤ 2hPG < 11.1 mmol/L), and newly diagnosed diabetes ([1] without a self-reported diagnosis of diabetes that was determined previously by a health care professional, and [2] FPG ≥ 7.0 mmol/L and/or 2hPG ≥ 11.1 mmol/L). We also used the standards of medical care in diabetes-2018 proposed by American Diabetes Association (ADA) to define newly diagnosed diabetes in a sensitivity analysis: participants without a history of diabetes who had  FPG ≥ 7.0 mmol/L or 2hPG ≥ 11.1 mmol/L during the baseline OGTT or HbA1c ≥ 6.5% [12].

### Statistical analysis

Sex-specific mean (SD) and frequency (proportion) were revealed by different diabetes status (Non-diabetes and newly diagnosed diabetes). Student’s t test and Chi square tests were used to assess differences between two groups for continuous and categorical variables, respectively (Table 1). Logistic regression was used to assess the associations of SFA and VFA with the risk of newly diagnosed diabetes. SFA and VFA were entered in the following two ways: (1) as quartiles, and (2) as a continuous variable. Four models were constructed: Model 1 adjusted for age; Model 2 adjusted for potential confounding factors: age, education, leisure-time physical activity, smoking habit, alcohol consumption, systolic blood pressure, and family history of diabetes; Model 3 took general obesity into account and adjusted for variables in Model 2 as well as BMI; model 4 adjusted for variables in Model 3 as well as VFA or SFA. The interaction between SFA and VFA was assessed by likelihood ratio test. The receiver operating characteristic (ROC) curve was plotted and area under the curve (AUC) was calculated to assess the ability of different adiposity indicators to discriminate newly diagnosed diabetes. Restricted cubic spline was nested in logistic models to further explore the nonlinear or dose–response association of SFA or VFA with the risk of newly diagnosed diabetes [13, 14]. In order to lessen the influence of extreme values, we included participants with SFA and VFA from the 0.5 to 99.5 percentiles (SFA [cm^2^] 33.0–270.1 and 47.0–352.1, VFA [cm^2^] 19.5–294.4 and 27.3–243.6 for men and women, respectively). Three knots were created for men (SFA [cm^2^] 54.5, 122.4, and 202.8; VFA [cm^2^] 42.4, 117.0, and 209.8) and women (SFA [cm^2^] 84.2, 161.3, and 268.5; VFA [cm^2^] 50.3, 103.5, and 179.9). All statistical analyses were performed with IBM SPSS Statistics for Windows, version 24.0 (IBM Corp, 2016) or SAS version 9.4 (SAS Institute Inc., Cary, NC, USA). *P* value < 0.050 (two-tailed) was considered statistically significant.

**Table 1:** General characteristics of study participants with and without newly diagnosed diabetes

Variables	Men			Women		
	Non-diabetes (*n* = 4614)	Newly diagnosed diabetes (*n* = 787)	*P* for differences^a^	Non-diabetes (*n* = 5734)	Newly diagnosed diabetes (*n* = 1002)	*P* for differences^a^
Age (years)	56.6 (6.5)	57.6 (6.4)	< 0.001	56.3 (6.5)	58.4 (6.4)	< 0.001
Fasting plasma glucose (mmol/L)	5.7 (0.5)	7.7 (2.1)	< 0.001	5.7 (0.5)	7.3 (1.8)	< 0.001
2-h plasma glucose (mmol/L)	6.9 (1.7)	13.6 (4.3)	< 0.001	7.4 (1.6)	13.8 (3.9)	< 0.001
Body mass index (kg/m^2^)	24.7 (3.0)	25.9 (3.1)	< 0.001	24.6 (3.2)	25.9 (3.5)	< 0.001
Waist circumference (cm)	86 (9)	89 (9)	< 0.001	82 (9)	85 (9)	< 0.001
Body fat percentage (%)	22.1 (5.1)	24.3 (4.8)	< 0.001	34.5 (5.5)	36.7 (5.8)	< 0.001
Systolic blood pressure^b^ (mmHg)	131 (14)	136 (15)	< 0.001	130 (15)	137 (15)	< 0.001
Diastolic blood pressure^b^ (mmHg)	84 (8)	86 (8)	< 0.001	82 (9)	85 (8)	<0.001
VFA (cm^2^)	116.8 (52.0)	145.6 (55.4)	< 0.001	104.2 (39.5)	128.3 (45.4)	< 0.001
SFA (cm^2^)	123.2 (46.2)	136.7 (47.2)	< 0.001	166.0 (57.0)	173.6 (60.7)	< 0.001
VFA/SFA	0.97 (0.36)	1.11 (0.41)	< 0.001	0.67 (0.28)	0.80 (0.33)	< 0.001
Levels of education, n (%)^c^			0.65			< 0.001
Primary school or less	1864 (40.9)	326 (42.4)		2989 (52.7)	635 (64.2)	
Middle school	2211 (48.6)	369 (48.0)		2234 (39.4)	295 (29.8)	
High school or more	478 (10.5)	74 (9.6)		450 (7.9)	59 (6.0)	
Smoking habit, n (%)			0.001			0.49
Never smoker	1913 (41.5)	363 (46.1)		5718 (99.7)	1001 (99.9)	
Ex-smoker	259 (5.6)	61 (7.8)		7 (0.1)	0	
Current smoker	2442 (52.9)	363 (46.1)		9 (0.2)	1 (0.1)	
Alcohol consumption, n (%)			0.129			0.003
Never drinker	2976 (64.5)	479 (60.9)		5702 (99.4)	995 (99.3)	
Ex-drinker	113 (2.4)	19 (2.4)		1 (0.02)	3 (0.3)	
Current drinker	1525 (33.1)	289 (36.7)		31 (0.5)	4 (0.4)	
Leisure-time physical activity, n (%)			0.85			0.66
0 min/day	4375 (94.8)	750 (95.3)		5386 (93.9)	945 (94.3)	
1–29 min/day	105 (2.3)	16 (2.0)		139 (2.4)	26 (2.6)	
≥ 30 min/day	134 (2.9)	21 (2.7)		209 (3.6)	31 (3.1)	
Family history of diabetes, n (%)^d^	559 (12.1)	133 (16.9)	< 0.001	709 (12.4)	227 (22.7)	< 0.001
Glucose regulation status			–			–
Normal glucose regulation	2576 (55.8)	–		3082 (53.7)	–	
Isolated impaired fasting glucose	630 (13.7)	–		502 (8.8)	–	
Isolated impaired glucose tolerance	927 (20.1)	–		1503 (26.2)	–	
Combined impaired fasting glucose and impaired glucose tolerance	481 (10.4)	–		647 (11.3)	–	

Data are presented as mean (SD), or frequency (percentage). *BMI* body mass index, *SFA* subcutaneous fat area, and *VFA* visceral fat area

^a^*P* for difference was calculated with the use of the Chi square test, and Mann–Whitney *U* for the comparison of participants with and without newly diagnosed diabetes

^b^Missing data: n = 2 (0.04%) in men without diabetes

^c^Missing data: n = 61 (1.3%) in men without diabetes, n = 18 (2.3%) in men with newly diagnosed diabetes, n = 61 (1.1%) in women without diabetes, n = 13 (1.3%) in women with newly diagnosed diabetes

^d^Missing data: n = 4 (0.07%) in women without diabetes, n = 1 (0.10%) in women with newly diagnosed diabetes

## Results

### Participants characteristics

General characteristics of the study population at baseline were presented in Table 1. A total of 5401 men and 6736 women were available for analysis. Participants of both sexes who were newly diagnosed with diabetes at baseline were slightly older, their FPG, 2hPG, BMI, waist circumference, blood pressure, SFA, VFA, and VFA/SFA were higher, and they were more likely to have a family history of diabetes, as compared with those who remained free of diabetes at baseline.

### Correlations between adiposity indicators

Age-adjusted partial correlations were 0.779 in men (*P *< 0.001) and 0.677 in women (*P *< 0.001) for BMI and SFA, 0.695 in men (*P *< 0.001) and 0.663 in women (*P *< 0.001) for BMI and VFA, 0.725 in men (*P *< 0.001) and 0.623 in women (*P *< 0.001) for waist circumference and SFA, and 0.696 in men (*P *< 0.001) and 0.624 in women (*P *< 0.001) for waist circumference and VFA (Additional file 2).

### Associations of SFA and VFA with risk of newly diagnosed diabetes

**Fig. 1:**
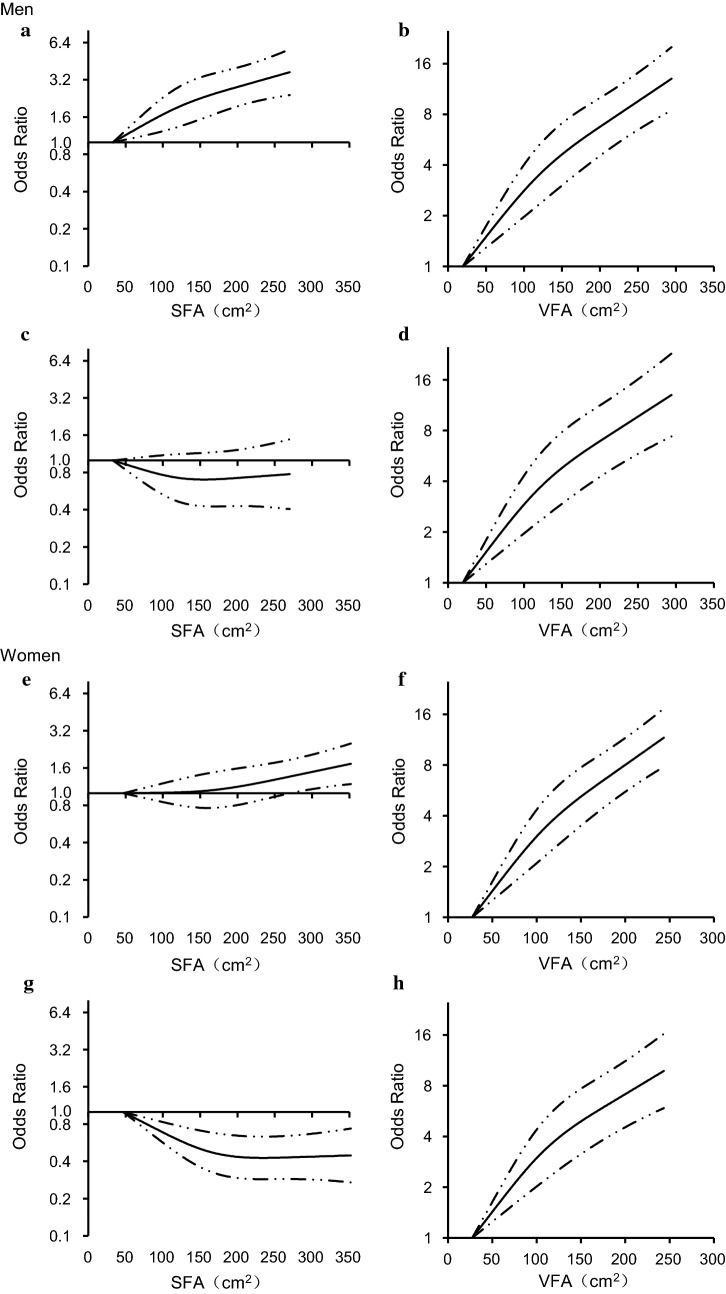
Odds ratios for newly diagnosed diabetes based on different levels of SFA and VFA. Solid lines are multivariable-adjusted odds ratios, and dashed lines indicate 95% confidence intervals derived from restricted cubic spline regression. Odds ratios are plotted on a log scale. Odds ratios of SFA and VFA for newly diagnosed diabetes after adjustment for age, level of education, smoking habit, alcohol consumption, leisure-time physical activity, systolic blood pressure, and family history of diabetes (Model 2) are presented in **a**, **b** for men, and **e**, **f** for women, respectively. Odds ratios of SFA and VFA for newly diagnosed diabetes after adjustment for variables in Model 2 and also for body mass index as well as VFA or SFA (Model 4) are presented in **c**, **d** for men, and **g**, **h** for women, respectively. *BMI* body mass index, *SFA* subcutaneous fat area, and *VFA* visceral fat area

The multivariable-adjusted (age, education, leisure-time physical activity, smoking habit, alcohol consumption, systolic blood pressure, and family history of diabetes—Model 2) odds ratios (OR) of newly diagnosed diabetes across quartiles of SFA were 1.00 (95% confidence interval [CI], 1.00–1.00), 1.52 (95% CI, 1.19–1.94), 1.75 (95% CI, 1.38–2.23), and 1.87 (95% CI, 1.48–2.38) (*P* for trend < 0.001) for men, and 1.00 (95% CI, 1.00–1.00), 1.06 (95% CI, 0.87–1.30), 1.07 (95% CI, 0.87–1.31), and 1.27 (95% CI, 1.05–1.55) (*P* for trend = 0.014) for women, respectively (Table 2). The association was non-significant (*P *= 0.261) in men after further adjustment for BMI (Model 3) and was reversed significantly after additional adjustment for VFA (Model 4) in men (*P* for trend= 0.042) and women (*P* for trend= 0.003). When SFA was examined as a continuous variable, the multivariable-adjusted (Model 2) ORs of newly diagnosed diabetes for each 1 SD increase in SFA were 1.29 (95% CI, 1.19–1.39) in men, and 1.10 (95% CI, 1.03–1.18) in women. When BMI, VFA and SFA were entered into the multivariable-adjusted model simultaneously (Model 4), the positive association disappeared in men and was reversed in women (OR 0.86 [95% CI, 0.78–0.94]). Restricted cubic spline models showed the positive association between SFA and the risk of newly diagnosed diabetes in multivariable-adjusted Model 2, and the inverse association between SFA and the risk of newly diagnosed diabetes in multivariable-adjusted Model 4 (Fig. 1a, c, e and g).

**Table 2:** Odds ratios of newly diagnosed diabetes based on SFA as a continuous or category variable

	No. of participants	No. of cases	Odds ratios (95% CI)^a^			
			Model 1^b^	Model 2^c^	Model 3^d^	Model 4^e^
Men						
SFA as categories						
Quartile 1	1348	129	1.00 (1.00–1.00)	1.00 (1.00–1.00)	1.00 (1.00–1.00)	1.00 (1.00–1.00)
Quartile 2	1351	194	1.62 (1.28–2.05)	1.52 (1.19–1.94)	1.14 (0.89–1.48)	0.98 (0.76–1.27)
Quartile 3	1350	225	1.94 (1.54–2.44)	1.75 (1.38–2.23)	1.10 (0.83–1.44)	0.90 (0.68–1.19)
Quartile 4	1352	239	2.12 (1.69–2.68)	1.87 (1.48–2.38)	0.88 (0.64–1.22)	0.74 (0.54–1.03)
* P* for trend			< 0.001	<0.001	0.261	0.042
SFA as a continuous variable (1 SD increase)			1.35 (1.25–1.45)	1.29 (1.19–1.39)	0.99 (0.88–1.12)	0.96 (0.85–1.09)
Women						
SFA as categories						
Quartile 1	1683	227	1.00 (1.00–1.00)	1.00 (1.00–1.00)	1.00 (1.00–1.00)	1.00 (1.00–1.00)
Quartile 2	1683	236	1.10 (0.90–1.34)	1.06 (0.87–1.30)	0.85 (0.69–1.04)	0.79 (0.64–0.98)
Quartile 3	1686	244	1.15 (0.94–1.40)	1.07 (0.87–1.31)	0.73 (0.59–0.91)	0.70 (0.56–0.88)
Quartile 4	1684	295	1.42 (1.17–1.71)	1.27 (1.05–1.55)	0.66 (0.52–0.84)	0.68 (0.53–0.87)
*P* for trend			< 0.001	0.014	0.001	0.003
SFA as a continuous variable (1 SD increase)			1.15 (1.08–1.23)	1.10 (1.03–1.18)	0.83 (0.76–0.91)	0.86 (0.78–0.94)

^a^The cut-off values of SFA quartiles are 92.6, 122.4, and 153.9 cm^2^ in men and 126.8, 161.2, and 201.2 cm^2^ in women. *SFA* subcutaneous fat area, and *VFA* visceral fat area

^b^Model 1 adjusted for age

^c^Model 2 adjusted for age, level of education, smoking habit, alcohol consumption, leisure-time physical activity, systolic blood pressure, and family history of diabetes

^d^Model 3 adjusted for variables in Model 2 and also for body mass index

^e^Model 4 adjusted for variables in Model 3 and also for VFA

The multivariable-adjusted (Model 2) ORs of newly diagnosed diabetes across quartiles of VFA were 1.00 (95% CI, 1.00–1.00), 1.22 (95% CI, 0.93–1.61), 2.26 (95% CI, 1.75–2.90), and 3.40 (95% CI, 2.66–4.34) (*P* for trend < 0.001) for men, and 1.00 (95% CI, 1.00–1.00), 1.65 (95% CI, 1.29–2.13), 2.32 (95% CI, 1.83–2.95), and 3.66 (95% CI, 2.90–4.61) (*P* for trend < 0.001) for women, respectively (Table 3). The positive association remained significant among men (*P* for trend< 0.001) and women (*P* for trend< 0.001) after further adjustment for BMI (Model 3) and SFA (Model 4). When VFA was examined as a continuous variable, the multivariable-adjusted (Model 4) ORs of newly diagnosed diabetes for each 1 SD increase in VFA were 1.58 (95% CI, 1.43–1.75) in men, and 1.49 (95% CI, 1.37–1.63) in women. Restricted cubic splines models showed the similar positive association between VFA as a continuous variable and the risk of newly diagnosed diabetes in multivariable-adjusted Model 2 and Model 4 in both sexes (Fig. 1b, d, f and h).

**Table 3:** Odds ratios of newly diagnosed diabetes based on VFA as a continuous or category variable

	No. of participants	No. of cases	Odds ratios (95% CI)^a^			
			Model 1^b^	Model 2^c^	Model 3^d^	Model 4^e^
Men						
VFA as categories						
Quartile 1	1349	102	1.00 (1.00–1.00)	1.00 (1.00–1.00)	1.00 (1.00–1.00)	1.00 (1.00–1.00)
Quartile 2	1346	133	1.34 (1.02–1.76)	1.22 (0.93–1.61)	1.14 (0.86–1.52)	1.17 (0.87–1.56)
Quartile 3	1353	229	2.54 (1.98–3.25)	2.26 (1.75–2.90)	2.04 (1.54–2.70)	2.10 (1.58–2.80)
Quartile 4	1353	323	3.89 (3.07–4.94)	3.40 (2.66–4.34)	2.92 (2.15–3.95)	2.99 (2.20–4.07)
*P* for trend			< 0.001	< 0.001	< 0.001	< 0.001
VFA as a continuous variable (1 SD increase)			1.68 (1.56–1.80)	1.61 (1.49–1.74)	1.58 (1.43–1.75)	1.58 (1.43–1.75)
Women						
VFA as categories						
Quartile 1	1681	110	1.00 (1.00–1.00)	1.00 (1.00–1.00)	1.00 (1.00–1.00)	1.00 (1.00–1.00)
Quartile 2	1681	192	1.78 (1.39–2.28)	1.65 (1.29–2.13)	1.58 (1.22–2.04)	1.62 (1.25–2.09)
Quartile 3	1689	274	2.59 (2.05–3.28)	2.32 (1.83–2.95)	2.13 (1.65–2.75)	2.18 (1.68–2.81)
Quartile 4	1685	426	4.29 (3.42–5.38)	3.66 (2.90–4.61)	3.19 (2.42–4.19)	3.17 (2.41–4.17)
*P* for trend			< 0.001	< 0.001	< 0.001	< 0.001
VFA as a continuous variable (1 SD increase)			1.64 (1.53–1.75)	1.56 (1.45–1.67)	1.51 (1.38–1.65)	1.49 (1.37–1.63)

^a^The cut-off values of VFA quartiles were 81.8, 117.0, and 154.8 cm^2^ in men and 78.3, 103.4, and 132.1 cm^2^ in women. *SFA* subcutaneous fat area, and VFA visceral fat area

^b^Model 1 adjusted for age

^c^Model 2 adjusted for age, level of education, smoking habit, alcohol consumption, leisure-time physical activity, systolic blood pressure, and family history of diabetes

^d^Model 3 adjusted for variables in Model 2 and also for body mass index

^e^Model 4 adjusted for variables in Model 3 and also for SFA

In a sensitivity analysis, the multivariable-adjusted associations of newly diagnosed diabetes (2018 ADA criteria) with SFA and VFA were presented in Additional file 3: Tables S1, S2. The odds ratios between newly diagnosed diabetes and SFA or VFA were comparable regardless of diabetes defined by ADA or WHO criteria.

Additional file 4 presented the ROC curve for predicted probability of newly diagnosed diabetes by different adiposity indicators (VFA, VFA/SFA, BMI, waist circumference, and body fat percentage) in multivariable-adjusted Model 2. The AUCs were 0.648–0.679 for men and 0.683–0.707 for women. The pairwise comparisons showed that the AUCs of newly diagnosed diabetes predicted by VFA (0.679 [95% CI, 0.659–0.699] for men and 0.707 [95% CI, 0.690–0.723] for women) were significantly larger than by the other adiposity indicators (All *P* values < 0.02). There were no difference among the AUCs of BMI, waist circumference, body fat percentage, and VFA/SFA.

**Fig. 2:**
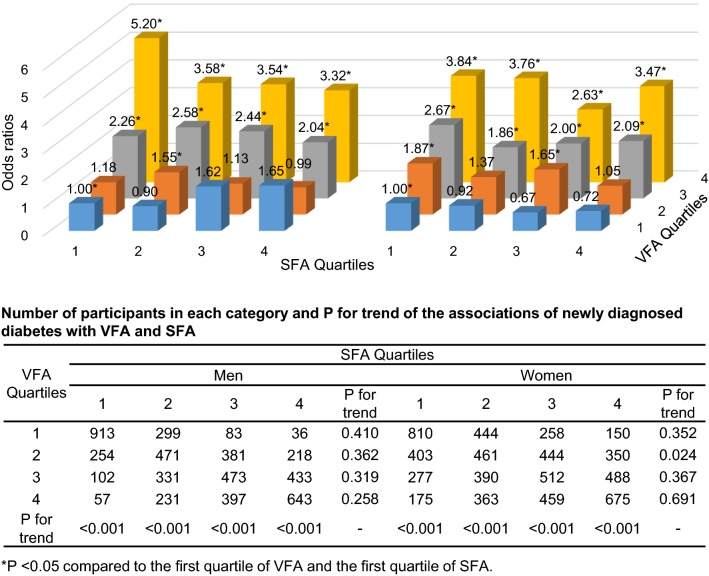
Joint association of VFA, SFA, and newly diagnosed diabetes. Odds ratios were calculated after adjustment for age, level of education, smoking habit, alcohol consumption, leisure-time physical activity, systolic blood pressure, and family history of diabetes. Number of participants in each category and P for trend of the association of newly diagnosed diabetes with VFA and SFA were listed in table. *SFA* subcutaneous fat area, and *VFA* visceral fat area

The joint associations of SFA and VFA with newly diagnosed diabetes risk in the multivariable-adjusted Model 2 were shown in Fig. 2. The positive associations between VFA and newly diagnosed diabetes risk were observed in participants with any quartile of SFA (All P for trend < 0.001). However, there were few significantly associations between SFA and newly diagnosed diabetes risk in participants with different quartiles of VFA. In women with VFA within the second quartile, SFA were negatively associated with risk of newly diagnosed diabetes (P for trend = 0.024). There were no significant interactions of VFA and SFA with the risk of newly diagnosed diabetes among both men and women (*P* values > 0.050).

## Discussion

The present study found that SFA measured by MRI was negatively associated with risk of newly diagnosed diabetes in women (OR 0.86 [95% CI, 0.78–0.94]) but not in men (OR 0.96 [95% CI, 0.85–1.09]), after taking general obesity (assessed by BMI) and visceral obesity (assessed by VFA) into account. However, VFA was positively associated with the risk of newly diagnosed diabetes among both Chinese men and women, independent of BMI and SFA. Among the obesity indices of VFA, VFA/SFA, BMI, waist circumference, and body fat percentage, VFA had the best ability (AUC 0.679 [95% CI, 0.659–0.699] for men and 0.707 [95% CI, 0.690–0.723] for women) to discriminate newly diagnosed diabetes.

Few studies have examined the association between subcutaneous adipose tissue measured by CT or MRI and the risk of diabetes, and the results are inconsistent. Fox et al. [3] and Liu et al. [4] elucidated a positive association between subcutaneous adipose tissue and diabetes risk in white and African Americans, but they did not adjust for BMI in their analyses [3, 4, 15]. In the SABRE Study, Eastwood et al. used anthropometry-based prediction equations to estimate baseline subcutaneous adipose tissue and found a positive association of estimated subcutaneous adipose tissue with the risk of diabetes after adjustment for BMI, but only in South Asian men, not in European men and women, South Asian women, or African Caribbean men and women [16]. Other studies, however, failed to find any associations of subcutaneous adipose tissue with the risk of diabetes [5, 7]. Several studies indicated that subcutaneous adipose tissue possessed protective properties of insulin sensitivity after adjustment for BMI and visceral adipose tissue [17] or waist circumference as well as body fat [18]. However, to the best of our knowledge, no human study has provided direct evidence of a negative association between subcutaneous adipose tissue and risk of diabetes. The present study found a positive association between SFA measured by MRI and the risk of newly diagnosed diabetes after adjustment for major risk factors of diabetes. However, this positive association was non-significant in men and was reversed significantly after additional adjustment for general obesity (assessed by BMI) and visceral obesity (assessed by VFA) in women. These results first indicated that SFA was a favorable adipose depot rather than a pathogenic adipose depot for diabetes in women, and BMI and VFA confound the association between SFA and diabetes.

The exact mechanism of the favorable effects of subcutaneous adipose tissue on diabetes risk remained unclear, but several possible mechanisms have been proposed. The secretion or expression of more favorable adipokines such as leptin and adiponectin was higher in subcutaneous adipose tissue than in visceral adipose tissue [19, 20]. In addition, it is documented in animal studies that transplantation of subcutaneous adipose tissue to the visceral cavity improved glucose tolerance and insulin sensitivity [21, 22]. It was hypothesized that when subcutaneous adipose failed to store excess energy, energy can alternatively be deposited in visceral adipose, the liver, or the heart [2, 23]. Acting like a lipid-buffering tissue, subcutaneous adipose may directly or indirectly act to maintain the homeostasis of metabolism.

Compared to deposits in other fat tissues, visceral adipose tissue was more metabolically active due to different levels and affinity of receptors related to lipolysis [24, 25] and activity of enzymes related to lipolysis [26, 27]. Excess visceral adipose tissue was correlated with higher levels of free fatty acid overflow [2], inflammatory markers [28–31], and adipocytokines [32–34]. Kim et al. reported that fatty acid binding protein 4, S100 calcium binding protein A8/9, SH3 domain containing 1, α-2-HS-glycoprotein, and complement component 1, q subcomponent, A chain were up-regulated, while perilipin 1 and 4, carnitine palmitoyltransferase 2, acyl-CoA dehydrogenase, long chain, and acyl-CoA dehydrogenase, C-4 to C-12 straight chain were down-regulated in visceral adipose tissue of subjects with type 2 diabetes than in visceral adipose tissue of those with normal glucose tolerances. These proteins were either metabolic enzymes, adipokines, or were involved in the inflammation related process [35], which may contribute to the progress of insulin resistance or even diabetes.

Visceral adipose tissue measured by CT had a positive association with diabetes risk, even when accounting for other adiposity indicators, such as BMI, in white Americans [3], African Americans [4], and Japanese Americans [5]. Unlike white and African Americans, Asians are more likely to have abdominal adipose tissue, which leads to an increased risk of type 2 diabetes mellitus at lower BMI [9, 26]. Only one recent Chinese study demonstrated that VFA was independently associated with an increased risk of diabetes in women but not in men [36]. However, the VFA was measured by a fat area analyzer using bioelectrical impedance analysis, which is a less accurate measure of body composition than MRI and dual-energy X-ray absorptiometry. Moreover, in most of the above mentioned studies diabetes was diagnosed by fasting glucose alone, not an OGTT. Therefore, the authors could have missed some cases of diabetes [37], which usually resulted in a biased estimate of the association between different adiposity and the risk of diabetes. In the present study, we used MRI to measure body composition, and used an OGTT to diagnose diabetes among more than 12,000 Chinese participants. We first found a graded positive association between VFA and the risk of newly diagnosed diabetes among both Chinese men and women, and this association was independent of BMI, SFA and other known risk factors of diabetes. Moreover, out of the five adiposity indicators (VFA, VFA/SFA, BMI, waist circumference, and body fat percentage), VFA possessed the best ability to discriminate newly diagnosed diabetes.

As a cardiometabolic disease, diabetes is associated with 2–4 times higher risk of CVD and it double the risk of cardiovascular mortality [38–41]. Previous studies have documented that ectopic fat accumulation, mainly visceral adipose tissue, acting through the potential inflammatory response and oxidative phosphorylation pathways [42], is a risk factor of metabolic disorders, atherosclerosis, and CVD [3, 43–45]. The failure of visceral adipose tissue to store triglyceride could result in an increase of epicardial adipose tissue thickness [46]. Epicardial adipose tissue may turn into an adverse lopotoxic and pro-inflammatory organ, discharge fat free acid into the blood, and lead to CVD [46]. In addition, some studies showed that liver fat increased risk of impaired glucose regulation and type 2 diabetes [47], and mid-thigh lipid-rich muscle was associated with higher carotid artery intima-media thickness [48]. Although the association of abdominal subcutaneous adipose tissue with risk of atherosclerosis and CVD was in dispute [49–52], most of studies showed that thigh fat possessed favorable effect on cardiometabolic diseases. Sophie et al. found that thigh subcutaneous adipose tissue was protective for diabetes [6]. Han et al. documented that higher leg fat to total fat ratio was associated with decreased risk of CVD assessed by atherosclerotic cardiovascular disease risk equations [53]. More evidence was needed to elucidate the impact of adipose distribution on CVD.

There were several advantages of our study. Firstly, this is the first large population-based study focusing on the associations of different adipose distributions measured by MRI with the risk of newly diagnosed diabetes among Chinese adults. Secondly, diagnosis of diabetes was based on the WHO criteria [11] after a 2-h 75-g OGTT, which provided a comprehensive and accurate estimation of diabetes. Thirdly, the results of the present study were reliable, as almost all potential confounding factors had been adjusted. However, the present results were drawn from a cross-sectional study regarding the associations of SFA and VFA with the risk of diabetes, by which causal relationship cannot be inferred. A longitudinal study is still ongoing in the present study to identify the indicative power of future incident diabetes.

## Conclusions

In conclusion, SFA measured by MRI was beneficial for lower risk of newly diagnosed diabetes in women but was not associated with newly diagnosed diabetes in men, while VFA was a good indicator for likelihood of newly diagnosed diabetes among Chinese adults, independently of BMI and SFA.

## Additional files

**Additional file 1.** Participation flowchart.

**Additional file 2.** Age-adjusted partial correlations between adiposity indicators.

**Additional file 3.** Odds ratios of newly diagnosed diabetes according to the ADA criteria based on SFA or VFA as a continuous or category variable.

**Additional file 4.** The receiver operating characteristic curve of adiposity indicators and newly diagnosed diabetes.

**Additional file 5.** List of investigators.

## Abbreviations

2hPG: 2-h plasma glucose
ADA: American Diabetes Association
AUC: area under the curve
BMI: body mass index
CVD: cardiovascular disease
FPG: fasting plasma glucose
HbA1c: glycated hemoglobin A1c
MRI: magnetic resonance imaging
OGTT: oral glucose tolerance test
OR: odds ratio
CI: confidence interval
CT: computed tomography
ROC: receiver operating characteristic
SFA: subcutaneous fat area
VFA: visceral fat area
WHO: World Health Organization
