# Supplementary material for: Abdominal subcutaneous adipose tissue: a favorable adipose depot for diabetes?
Source: Cardiovasc Diabetol. 2018 Jun 26;17:93. doi: 10.1186/s12933-018-0734-8 (PMC6020307; doi:10.1186/s12933-018-0734-8)
Supplement: Supplementary file 1 — Additional file 1. Participation flowchart. [file 12933_2018_734_MOESM1_ESM.docx]

**Additional file 1. Participation flowchart**

All participants aged 45-70 years

N = 23,375

Participants finished survey

N = 17,212

Participants were enrolled

N = 21,408

Excluded 1,967

Could not be contacted 714

Rejected 1,253

Excluded 5,075

Missing BMI data 164

Missing waist circumference data 11

Missing body fat data 916

Missing fasting or 2-hour glucose data 75

Missing VFA and SFA data 2,861

Participants with known diabetes 1,048

Participants in the analysis

N = 12,137
