# Supplementary material for: Abdominal subcutaneous adipose tissue: a favorable adipose depot for diabetes?
Source: Cardiovasc Diabetol. 2018 Jun 26;17:93. doi: 10.1186/s12933-018-0734-8 (PMC6020307; doi:10.1186/s12933-018-0734-8)
Supplement: Supplementary file 2 — Additional file 2. Age-adjusted partial correlations between adiposity indicators. [file 12933_2018_734_MOESM2_ESM.docx]

**Additional file 2. Age-adjusted partial correlations between adiposity indicators^a^**

| **Variables** | **Men** | | | | **Women** | | | |
| --- | --- | --- | --- | --- | --- | --- | --- | --- |
|  | **VFA** | **SFA** | **BMI** | **Waist circumference** | **VFA** | **SFA** | **BMI** | **Waist circumference** |
| SFA | 0.604 | - | - | - | 0.418 | - | - | - |
| BMI | 0.695 | 0.779 | - | - | 0.663 | 0.677 | - | - |
| Waist circumference | 0.696 | 0.725 | 0.780 | - | 0.624 | 0.623 | 0.769 | - |
| Body fat percentage | 0.678 | 0.745 | 0.827 | 0.737 | 0.654 | 0.668 | 0.916 | 0.742 |

^a^*P* values of all correlations were less than 0.001. BMI = body mass index, SFA = subcutaneous fat area, and VFA = visceral fat area.
