# Supplementary material for: Abdominal subcutaneous adipose tissue: a favorable adipose depot for diabetes?
Source: Cardiovasc Diabetol. 2018 Jun 26;17:93. doi: 10.1186/s12933-018-0734-8 (PMC6020307; doi:10.1186/s12933-018-0734-8)
Supplement: Supplementary file 3 — Additional file 3. Odds ratios of newly diagnosed diabetes according to the ADA criteria based on SFA or VFA as a continuous or category variable. [file 12933_2018_734_MOESM3_ESM.docx]

**Additional file 3: Table S1. Odds ratios of newly diagnosed diabetes according to the ADA criteria based on SFA as a continuous or category variable^a^**

|  | No. of participants | No. of cases | Odds ratios (95% CI)^a^ | | | |
| --- | --- | --- | --- | --- | --- | --- |
|  |  |  | Model 1^b^ | Model 2^c^ | Model 3^d^ | Model 4^e^ |
| Men |  |  |  |  |  |  |
| SFA as categories |  |  |  |  |  |  |
| Quartile 1 | 1346 | 141 | 1.00 (1.00-1.00) | 1.00 (1.00-1.00) | 1.00 (1.00-1.00) | 1.00 (1.00-1.00) |
| Quartile 2 | 1350 | 201 | 1.53 (1.21-1.92) | 1.43 (1.13-1.82) | 1.08 (0.84-1.39) | 0.93 (0.72-1.20) |
| Quartile 3 | 1349 | 240 | 1.90 (1.52-2.38) | 1.73 (1.37-2.17) | 1.08 (0.83-1.41) | 0.89 (0.68-1.17) |
| Quartile 4 | 1352 | 267 | 2.21 (1.77-2.75) | 1.97 (1.56-2.47) | 0.93 (0.68-1.27) | 0.79 (0.58-1.08) |
| *P* for trend |  |  | <0.001 | <0.001 | 0.521 | 0.119 |
| SFA as a continuous variable (1 SD increase) |  |  | 1.38 (1.28-1.48) | 1.32 (1.23-1.43) | 1.03 (0.91-1.15) | 0.99 (0.88-1.12) |
| Women |  |  |  |  |  |  |
| SFA as categories |  |  |  |  |  |  |
| Quartile 1 | 1682 | 238 | 1.00 (1.00-1.00) | 1.00 (1.00-1.00) | 1.00 (1.00-1.00) | 1.00 (1.00-1.00) |
| Quartile 2 | 1677 | 256 | 1.15 (0.95-1.40) | 1.12 (0.92-1.37) | 0.90 (0.73-1.10) | 0.84 (0.69-1.04) |
| Quartile 3 | 1684 | 270 | 1.23 (1.02-1.49) | 1.16 (0.95-1.41) | 0.80 (0.65-0.99) | 0.77 (0.62-0.95) |
| Quartile 4 | 1681 | 310 | 1.43 (1.19-1.73) | 1.29 (1.07-1.56) | 0.68 (0.54-0.86) | 0.70 (0.55-0.89) |
| *P* for trend |  |  | <0.001 | 0.009 | 0.001 | 0.004 |
| SFA as a continuous variable (1 SD increase) |  |  | 1.15 (1.08-1.22) | 1.10 (1.03-1.17) | 0.83 (0.76-0.91) | 0.86 (0.78-0.94) |

^a^The cut-off values of SFA quartiles are 92.6 cm^2^, 122.4 cm^2^, and 153.9 cm^2^ in men and 126.8 cm^2^, 161.2 cm^2^, and 201.2 cm^2^ in women. ADA = American Diabetes Association, SFA = subcutaneous fat area, and VFA = visceral fat area.

^b^Model 1 adjusted for age;

^c^Model 2 adjusted for age, level of education, smoking habit, alcohol consumption, leisure-time physical activity, systolic blood pressure, and family history of diabetes;

^d^Model 3 adjusted for variables in model 2 and also for body mass index;

^e^Model 4 adjusted for variables in model 3 and also for VFA.

**Additional file 3: Table S2. Odds ratios of newly diagnosed diabetes according to the ADA criteria based on VFA as a continuous or category variable^a^**

|  | No. of participants | No. of cases | Odds ratios (95% CI)^a^ | | | |
| --- | --- | --- | --- | --- | --- | --- |
|  |  |  | Model 1^b^ | Model 2^c^ | Model 3^d^ | Model 4^e^ |
| Men |  |  |  |  |  |  |
| VFA as categories |  |  |  |  |  |  |
| Quartile 1 | 1348 | 108 | 1.00 (1.00-1.00) | 1.00 (1.00-1.00) | 1.00 (1.00-1.00) | 1.00 (1.00-1.00) |
| Quartile 2 | 1345 | 152 | 1.46 (1.13-1.90) | 1.34 (1.03-1.75) | 1.23 (0.93-1.62) | 1.24 (0.94-1.64) |
| Quartile 3 | 1351 | 243 | 2.57 (2.02-3.27) | 2.30 (1.79-2.94) | 2.02 (1.54-2.65) | 2.05 (1.55-2.70) |
| Quartile 4 | 1353 | 346 | 4.01 (3.18-5.06) | 3.53 (2.78-4.48) | 2.90 (2.16-3.9) | 2.94 (2.18-3.96) |
| *P* for trend |  |  | <0.001 | <0.001 | <0.001 | <0.001 |
| VFA as a continuous variable (1 SD increase) |  |  | 1.68 (1.56-1.81) | 1.62 (1.50-1.74) | 1.56 (1.41-1.72) | 1.56 (1.41-1.73) |
| Women |  |  |  |  |  |  |
| VFA as categories |  |  |  |  |  |  |
| Quartile 1 | 1678 | 120 | 1.00 (1.00-1.00) | 1.00 (1.00-1.00) | 1.00 (1.00-1.00) | 1.00 (1.00-1.00) |
| Quartile 2 | 1676 | 206 | 1.75 (1.38-2.22) | 1.63 (1.28-2.08) | 1.56 (1.22-2.00) | 1.60 (1.25-2.05) |
| Quartile 3 | 1687 | 292 | 2.53 (2.02-3.17) | 2.27 (1.80-2.86) | 2.11 (1.65-2.70) | 2.15 (1.68-2.75) |
| Quartile 4 | 1683 | 456 | 4.23 (3.40-5.26) | 3.62 (2.89-4.53) | 3.21 (2.46-4.18) | 3.19 (2.44-4.16) |
| *P* for trend |  |  | <0.001 | <0.001 | <0.001 | <0.001 |
| VFA as a continuous variable (1 SD increase) |  |  | 1.63 (1.53-1.74) | 1.55 (1.45-1.66) | 1.51 (1.39-1.65) | 1.50 (1.37-1.63) |

^a^The cut-off values of VFA quartiles were 81.8 cm^2^, 117.0 cm^2^, and 154.8 cm^2^ in men and 78.3 cm^2^, 103.4 cm^2^ , and 132.1 cm^2^ in women. ADA = American Diabetes Association, SFA = subcutaneous fat area, and VFA = visceral fat area.

^b^Model 1 adjusted for age;

^c^Model 2 adjusted for age, level of education, smoking habit, alcohol consumption, leisure-time physical activity, systolic blood pressure, and family history of diabetes;

^d^Model 3 adjusted for variables in model 2 and also for body mass index;

^e^Model 4 adjusted for variables in model 3 and also for SFA.
