# Supplementary material for: Abdominal subcutaneous adipose tissue: a favorable adipose depot for diabetes?
Source: Cardiovasc Diabetol. 2018 Jun 26;17:93. doi: 10.1186/s12933-018-0734-8 (PMC6020307; doi:10.1186/s12933-018-0734-8)
Supplement: Supplementary file 4 — Additional file 4. The receiver operating characteristic curve of adiposity indicators and newly diagnosed diabetes. [file 12933_2018_734_MOESM4_ESM.docx]

**Additional file 4. The receiver operating characteristic** **(ROC) curve of adiposity indicators and newly diagnosed diabetes.**

The ROC curves were plotted using adiposity indicators (BMI, waist circumference, body fat percentage, VFA, VFA/SFA) to discriminate newly diagnosed diabetes after adjustment for age, education, leisure-time physical activity, smoking habit, alcohol drinking consumption, systolic blood pressure, and family history of diabetes. BMI = body mass index, SFA = subcutaneous fat area, and VFA = visceral fat area.

0.25

0.5

0.75

1.0

0.25

0.5

0.75

1.0

Sensitivity

Sensitivity

0

0.25

0.5

0.75

1.0

0

0.25

0.5

0.75

1.0

0

0

1-specificity

1-specificity

Men

Women

**
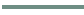

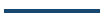

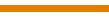

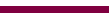

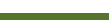
**

VFA/SFA

VFA

Body fat percentage

Waist circumference

BMI

| Adiposity indicators | Area Under Curve | P for difference |
| --- | --- | --- |
|  |  | vs. VFA |
| VFA | 0.679 (0.659-0.699) | - |
| VFA/SFA | 0.653 (0.633-0.674) | <0.001 |
| BMI | 0.650 (0.630-0.671) | <0.001 |
| Waist circumference | 0.648 (0.628-0.668) | <0.001 |
| Body fat percentage | 0.657 (0.637-0.677) | 0.001 |

| Adiposity indicators | Area Under Curve | P for difference |
| --- | --- | --- |
|  |  | vs. VFA |
| VFA | 0.707 (0.690-0.723) | - |
| VFA/SFA | 0.694 (0.677-0.711) | 0.015 |
| BMI | 0.686 (0.669-0.703) | <0.001 |
| Waist circumference | 0.685 (0.668-0.703) | <0.001 |
| Body fat percentage | 0.683 (0.666-0.701) | <0.001 |
