# Supplementary material for: Abdominal subcutaneous adipose tissue: a favorable adipose depot for diabetes?
Source: Cardiovasc Diabetol. 2018 Jun 26;17:93. doi: 10.1186/s12933-018-0734-8 (PMC6020307; doi:10.1186/s12933-018-0734-8)
Supplement: Supplementary file 5 — Additional file 5. List of investigators. [file 12933_2018_734_MOESM5_ESM.docx]

**LIST OF INVESTIGATORS**

**Dep****art****ment of Endocrinology and Metabolism, Shanghai Jiao Tong University Affiliated Sixth People’s Hospital:** Weiping Jia^ab^, MD, PhD, Yuqian Bao^ab^, MD, Xuhong Hou^a^, MD, PhD, Qingyi Sun, BS, Huijuan Lu, BS, Li Wei, MD, PhD, Peizhu Chen, MD, Jun Lu, MD, PhD.

**Shanghai Sixth People’s Hospital East Campus:** Mao Ye^b^, MD, Huaiyu Liu^b^, MD.

**Community Health Service Centre of Nicheng, Pudong District of Shanghai:** Musong Liu^b^, MD, Zhengxun Sun, BS, Jianming Wan, BS, Liping Gong, MD, Guiying Zhang, MD, Ying Ma, BS, Jianqiang Yang, BS, Yinguan Yan, BS, Lin Xia, BS, Chunjun Ma, MD, Chunhua Mao, MD, Keqing Dong, MD.

**Department of Radiology, Shanghai Jiao Tong University Affiliated Sixth People’s Hospital:** Chungen Wu, MD, PhD, Lei Jiao, MD, Hongmei Wang, BS, Songhua Li, MD, Yan Chen, MD, Yayan Shi, MD, Yue Du, MD.

**Computer center, Shanghai Jiao Tong University Affiliated Sixth People’s Hospital:** Yu Sun, ME, Yunzhen Wu, BE.

**Department of Ophthalmology, Shanghai Jiao Tong University Affiliated Sixth People’s Hospital:** Liang Yan, MD, PhD, Qiang Wu, MD, PhD, Lili Jia, MD, Xinhua Du, MD.

**Depa****rtment of Ultrasound in Medicine, Shanghai Jiao Tong University Affiliated Sixth People’s Hospital:** Fang Ma, MD, Weixing Zhang, MD, Yimin Jiang, MD, Liang feng, MD, Lichang Zhong, MD.

**Department of** **Nutrition, Shanghai Jiao Tong University Affiliated Sixth People’s Hospital:** Sheng Ge, MD, PhD, Wenguang Sun MD, PhD.

**Department of Laboratory, Shanghai Jiao Tong University Affiliated Sixth People’s Hospital:** Hua Liu, MD, Jing Xu, MD, Yingzhi Wang, MD, Yunyue Shen, MD, Qintao Zhang, BS, Xuhui Li, BS, Chunxu Zhang, BS, Ting Liu, BS, Weibin Chen, BS, Li Li, BS, Guo Wei, BS, Qinqing Liu, MD, Junling Tang, BS, Lu Dong, BS, Chenchen Gu, BS.

**Cardiac function room, Shanghai Jiao Tong University Affiliated Sixth People’s Hospital:** Weiguo Hu, MD, Jie Zhang, BS, Xin Chen, MD.

All the above investigators made substantial contributions to data collection.

^a^Also responsible for study concept and design.

^b^Also responsible for administrative, technical, or material support.
